# Supplementary material for: A Comprehensive Assessment of Ultraviolet-Radiation-Induced Mutations in Flammulina filiformis Using Whole-Genome Resequencing
Source: J Fungi (Basel). 2024 Mar 20;10(3):228. doi: 10.3390/jof10030228 (PMC10971301; doi:10.3390/jof10030228)
Supplement: Supplementary file 1 [file jof-10-00228-s001.zip › Supplementary Material S8/KEGG annotation/out/64381550635650.os/KO/out_map/map04623.html]

KEGG PATHWAY: Cytosolic DNA-sensing pathway - Reference pathway


|  |  |
| --- | --- |
| **Cytosolic DNA-sensing pathway - Reference pathway** |  |

[
Pathway menu
| Organism menu
| Pathway entry
| Show description
| User data mapping
]

Specific families of pattern recognition receptors are responsible for detecting foreign DNA from invading microbes or host cells and generating innate immune responses. DAI is the first identified sensor of cytosolic DNA which activates the IRF and NF-{kappa}B transcription factors, leading to production of type I interferon and other cytokines. The second type of cytoplasmic DNA sensor is AIM2. Upon sensing DNA, AIM2 triggers the assembly of the inflammasome, culminating in interleukin maturation. In addition to these receptors, there is a mechanism to sense foreign DNA, with the host RNA polymerase III converting the DNA into RNA for recognition by the RNA sensor RIG-I. These pathways provide various means to alert the cell.

|  |  |
| --- | --- |
| Reference pathway | 100% |
